# A retrospective study of kidney disease in Alport syndrome during and after pregnancy

**DOI:** 10.1007/s40620-025-02252-2

**Published:** 2025-05-20

**Authors:** Xinxin Kong, Jan Boeckhaus, Fang Wang, Chunyan Shi, Hongwen Zhang, Oliver Gross, Jie Ding, Yanqin Zhang

**Affiliations:** 1 Department of Pediatrics, Peking University First Hospital, No. 1 Xi An Men Da Jie, Beijing, 100034 China; 2 Clinic for Nephrology and Rheumatology, University Medicine Goettingen, Robert-Koch Str. 40, 37075 Goettingen, Germany; 3 Department of Gynaecology and Obstetrics, Peking University First Hospital, No. 1 Xi An Men Da Jie, Beijing, 100034 China

**Keywords:** Alport syndrome, Proteinuria, Pregnancy, Women

## Abstract

**Background:**

During pregnancy, hyperfiltration and other factors are hypothesized to contribute to the progression of kidney disease in women with Alport syndrome. To evaluate the status of kidney disease, clinical data from mothers with Alport syndrome in China and Europe over the pregnancy were analyzed.

**Methods:**

This retrospective observational study collected data to evaluate proteinuria, kidney function and Alport stage prior to, during, and after pregnancy, respectively.

**Results:**

A total of 74 women were enrolled, 82% of them with X-linked Alport syndrome and 11% with autosomal Alport syndrome (unknown in 5 patients). Detailed information on the course of pregnancy was available for 62 pregnancies from 52 different women. No fetal malformations were observed. Mean gestational age was 37.9 ± 2.7 weeks (*n* = 55).﻿ Complications included high blood pressure (*n* = 8), abortion (*n* = 5), preeclampsia (*n* = 5), gestational diabetes (*n* = 3), nephrotic syndrome (*n* = 2), cervical insufficiency with fetal growth delay (*n* = 2), premature rupture of membranes (*n* = 1) and acute intrauterine fetal distress (*n* = 1). Median proteinuria was 350 (30–2465) mg/day prior to pregnancy, 2390 (450–11,450) mg/day during pregnancy, and 590 (40–2650) mg/day at a mean postpartum follow-up time of 4.5 ± 2.1 years. Mean estimated glomerular filtration rate (eGFR) decreased by 17.2 ± 16.7 ml/min/1.73 m^2^, from 96.1 ± 32.9 to 78.9 ± 37 ml/min/1.73 m^2^ after pregnancy (*n* = 15; *p* = 0.003). The eGFR loss was higher in women with eGFR < 90 ml/min/1.73 m^2^ prior to pregnancy compared to women with normal renal function (– 21.5 ± 9.8 vs. – 14 ± 20 ml/min/1.73 m^2^), and in women with severe variants compared to women with less severe variants (– 21.5 ± 20.2 vs. – 11.3 ± 19.0 ml/min/1.73 m^2^). Progression of Alport stage after pregnancy was observed in 42% of the women, 31% remained in stage 0-1, and 23% remained in stage 2.

**Conclusions:**

This study provides important data on the natural history of Alport syndrome in women who have undergone  a pregnancy. Women with severe variants of Alport syndrome, and women with eGFR below 90 ml/min/1.73 m^2^ face greater risks of kidney disease progression after pregnancy. Further prospective studies are required to confirm these findings.

**Graphical abstract:**

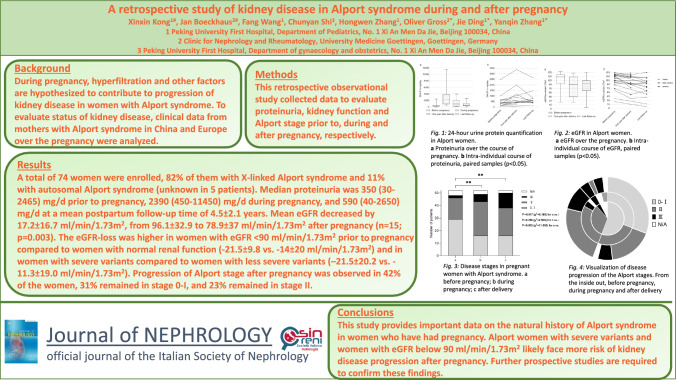

**Supplementary Information:**

The online version contains supplementary material available at 10.1007/s40620-025-02252-2.

## Introduction

Pregnant women with chronic kidney disease (CKD) have an elevated risk of adverse pregnancy-related outcomes [1]. The risks include preeclampsia, premature delivery, miscarriage, and delayed fetal growth. These complications correlate with severity of CKD proteinuria and hypertension [1, 2].

In Alport syndrome (AS), the most common hereditary glomerular kidney disease, most affected women develop microalbuminuria or proteinuria, and many develop impaired kidney function late in life [3, 4]. The number of women with Alport syndrome and pregnancy reported in the literature is limited and data about maternal outcomes in Alport syndrome are sparse [4].

Alport syndrome is caused by pathogenic variants in *COL4A3, COL4A4,* and *COL4A5* genes encoding the α3, α4, and α5 chains of collagen type IV, respectively [5]. It is clinically characterized by hematuria, proteinuria, and progressive kidney failure, as well as hearing loss and ocular abnormalities [6]. The X-linked form of Alport syndrome is caused by variants in the *COL4A5* gene [7], and the autosomal form is caused by variants in *COL4A3* or *COL4A4* [8]. Females with autosomal recessive Alport syndome have a high risk of early onset kidney failure (KF) [9–12]. In contrast, females with X-linked Alport syndrome usually have a  milder clinical disease course [13–15]. By the age of 40, 12–15% of women with X-linked Alport syndrome reach kidney failure, and 15% to 30% are reported to have kidney failure by the age of 60 [16].

Recent international Alport workshops have revealed a significant unmet medical need in clinical research on the risk to mother and child during pregnancy in patients with Alport syndrome [3, 17]. There is little information on whether pregnancy affects kidney impairment in women with Alport syndrome [2, 3]. A limited number of case reports point to a risk of accelerated kidney function impairment after delivery [18–20]. In order to address this knowledge gap, we conducted an observational, retrospective study on pregnant women with Alport syndrome and merged data from two cohorts from China and Europe. The combination of cohorts from China and Europe enabled the inclusion of a relatively high number of patients (*n* = 74) and pregnancies (*n* = 85) in the current analysis.

## Materials and methods

### Patients

Women with Alport syndrome were retrospectively selected from the Peking University First Hospital and the European Alport Registry in Goettingen. The Chinese cohort included women with Alport syndrome who had a history of pregnancy and were followed at Peking University First Hospital from 2011 to 2021. The European cohort includes patients with Alport syndrome who sent their data regarding the course of pregnancy and kidney function during or after pregnancy to the European Alport registry in Goettingen.

The diagnostic criteria for Alport syndrome [5, 13] included clinical manifestations such as hematuria, proteinuria, kidney impairment, progressive sensorineural hearing loss, or characteristic ocular lesions (lenticonus or maculopathy), and any of the following: (i) heterozygous pathogenic variants in the *COL4A5* gene for X-linked Alport syndrome; (ii) homozygous or compound heterozygous pathogenic variants in the *COL4A3* or *COL4A4* gene for autosomal recessive Alport syndome; (iii) kidney biopsy with typical changes of Alport syndrome, like splitting or lamellation in glomerular basement membrane (GBM); (iv) family history of Alport syndrome. Patients with an uncertain diagnosis of Alport syndrome were excluded.

The clinical data prior to, during, and after pregnancy were retrospectively collected and analyzed, including age, height, weight, number of deliveries, hematuria, proteinuria, kidney function, genetic testing, prenatal diagnosis, complications, fetal outcome, treatment, and family history.

### Groups

Alport syndrome stages were defined according to previous reports [21, 22] as follows: stage 0-I: microhematuria and/or microalbuminuria; stage II: overt proteinuria (proteinuria > 0.15 g/day); stage III: impaired kidney function [23] [S1-S2] (prior to, and after, pregnancy: estimated glomerular filtration rate [eGFR] < 90 ml/min/1.73 m^2^; during pregnancy: serum creatinine > 0.87 mg/dl or 77 µmol/l).

High blood pressure was diagnosed when the blood pressure level was ≥ 140 mm Hg systolic blood pressure (SBP) or ≥ 90 mm Hg diastolic blood pressure (DBP).

According to the pathogenic variants detected in the *COL4A5, COL4A3* or *COL4A4* genes, large deletions, nonsense variants, frameshifts, and splicing variants were defined as severe variants, whereas missense variants were defined as less severe variants [24].

### Statistical analysis

SPSS 27 was used for statistical analysis. Medians and ranges or means and standard deviation were used for continuous variables. Mann–Whitney test and Wilcoxon signed rank test were used to compare the continuous variables in different groups. Frequencies and percentages were used for categorical variables. Probability values (*p* values) below 0.05 were considered statistically significant.

## Results

### Patient characteristics

Altogether, 74 women with 85 pregnancies were included (52 from China and 22 from Europe). Mode of inheritance was X-linked in 61 women (82.4%), autosomal recessive in eight (10.8%) and unknown in five women (6.8%). Of the 61 women with X-linked Alport syndrome, 30 (49.2%) had a severe disease-causing variant, 26 (42.6%) had a less severe disease-causing variant, while the severity of the variant was unknown in 5 women (8.2%). Of the eight women with autosomal recessive Alport syndome, two had severe variants, one had two less severe variants, and the severity of the variant was unknown in two homozygous women. The three remaining women with autosomal Alport syndrome were heterozygous, with one severe, one less severe and one variant of unknown severity. One patient with autosomal recessive Alport syndome had been previously reported as a case report [25].

Mean age at first pregnancy was 31 ± 4.6 years (*n* = 74). Median Body Mass Index (BMI) prior to pregnancy was 21.8 kg/m^2^ (range 16.8–31.2 kg/m^2^) (*n* = 51). Median proteinuria was 350 mg/day (range 30–2465 mg/day) (*n* = 17) and mean eGFR was normal at 105.4 ± 30.7 ml/min/1.73 m^2^ (*n* = 22) at a median time of 0.4 years (range 0.1–3.9 years) before pregnancy.

Detailed information on the course of pregnancy was available for 62 pregnancies from 52 women. All patients discontinued renin–angiotensin–aldosterone system (RAAS) blockers prior to, or during the first month of pregnancy; no fetal malformations were observed. Mean gestational age was 37.9 ± 2.7 weeks (*n* = 55). Patients’ mean weight gain during pregnancy was 13 ± 3.9 kg (*n* = 21). Mean blood pressure was similar at the beginning and at the end of the pregnancies (119/75 ± 11/11.8 vs 118.8/76.1 ± 13.6/11.7 mmHg, *n* = 22). An event-free, uncomplicated pregnancy was reported in 48 out of 62 pregnancies (77.4%). Complications included high blood pressure (*n* = 8), abortion (*n* = 5), preeclampsia (*n* = 5), gestational diabetes (*n* = 3), nephrotic syndrome (*n* = 2), cervical insufficiency with fetal growth delay (*n* = 2), premature rupture of membranes (*n* = 1) and acute intrauterine fetal distress (*n* = 1). One of the women with nephrotic syndrome developed polyhydramnios. One woman with preeclampsia had an acute on chronic kidney injury during acute appendicitis that required kidney replacement therapy due to volume overload and lung edema. She also developed peripartum cardiomyopathy.

In addition, prenatal genetic diagnosis was performed in 37 women with X-linked Alport syndrome; 16 fetuses (43%) showed pathogenic variants (10 heterozygotes, 6 hemizygotes), and 13 of the 16 women decided to terminate their pregnancy.

### Proteinuria prior to, during, and after pregnancy

**Fig. 1:**
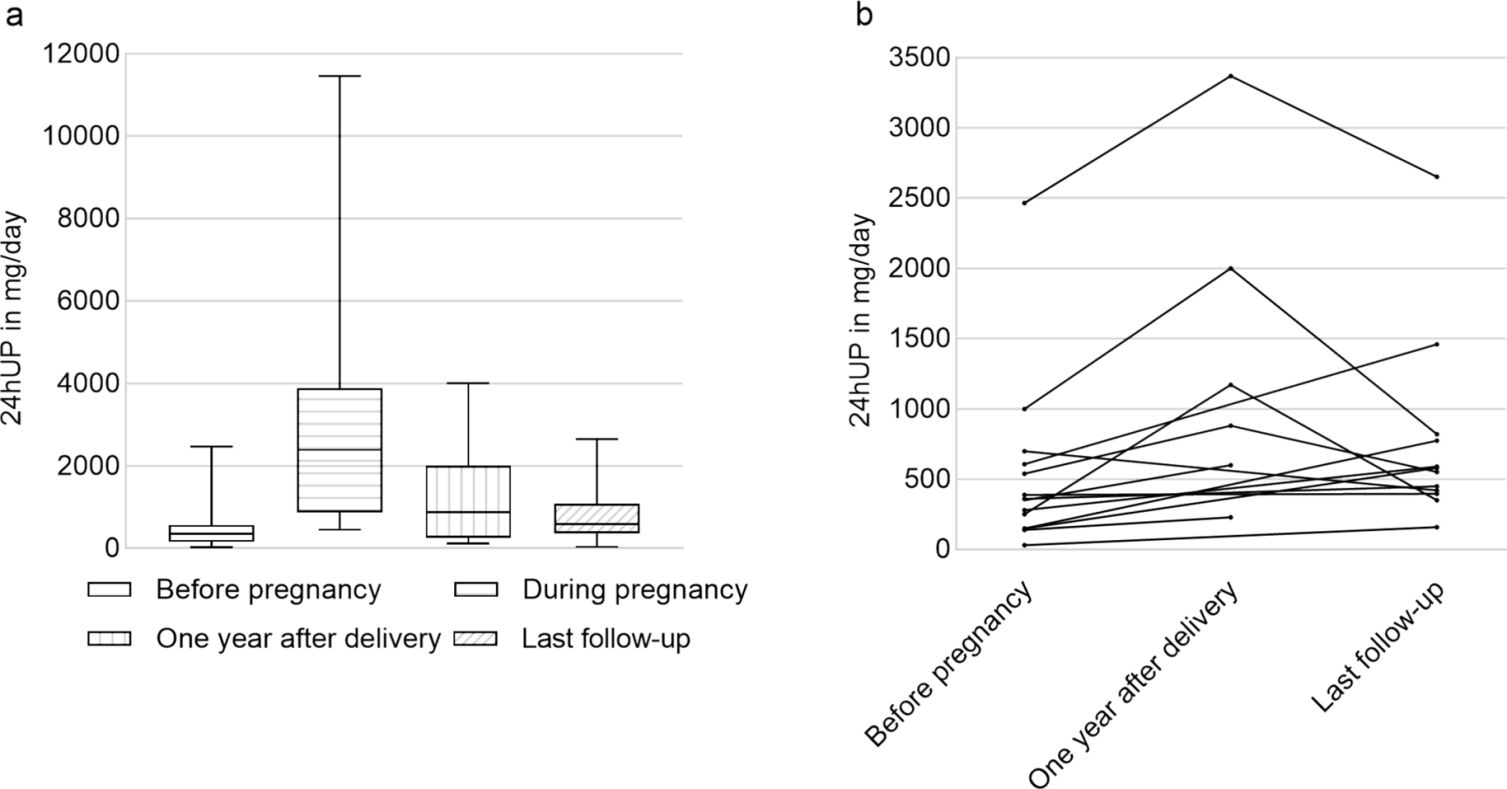
24-h urine protein quantification in women before and after pregnancy. **a** Proteinuria before pregnancy (*n* = 17; median 350 mg/day, range 30–2465 mg/day), during pregnancy (*n* = 7; median 2390 mg/day, range 450–11450 mg/day), one year after delivery (*n* = 13; median 880 mg/day, range 110–4000 mg/day) and last follow-up (*n* = 21; median 590 mg/day, range 40–2650 mg/day; median 5 years, range 1–9 years after delivery). **b** Intra-individual course of proteinuria, paired samples, Wilcoxon test (p < 0.05)

Data concerning proteinuria before pregnancy were available for 17 women with heterozygous X-linked Alport syndrome, during pregnancy for 7 women (one with autosomal-recessive and 6 with heterozygous X-linked Alport syndrome), and after pregnancy for 26 women (two with autosomal-recessive and 24 with heterozygous X-linked Alport syndrome) (Fig. 1a). For paired samples, compared to those before pregnancy, proteinuria at one year after delivery increased significantly from 445 (140–2465) mg/d to 1025 (230–3367) mg/day (*n* = 6; *p* = 0.028). At a mean follow-up time of 4.5 ± 2.1 years after delivery, median level of 24-h urine protein quantification was still higher than that before pregnancy, which increased from 375 (30–2465) mg/d to 565 (158–2650) mg/day (*n* = 12; *p* = 0.05) (Fig. 1b). Compared with proteinuria before pregnancy, a significant increase in proteinuria was observed in 50% of the women (7/14) at one year after delivery and at last follow-up (Fig. 1b).

### Kidney function prior to, during, and after pregnancy

**Fig. 2:**
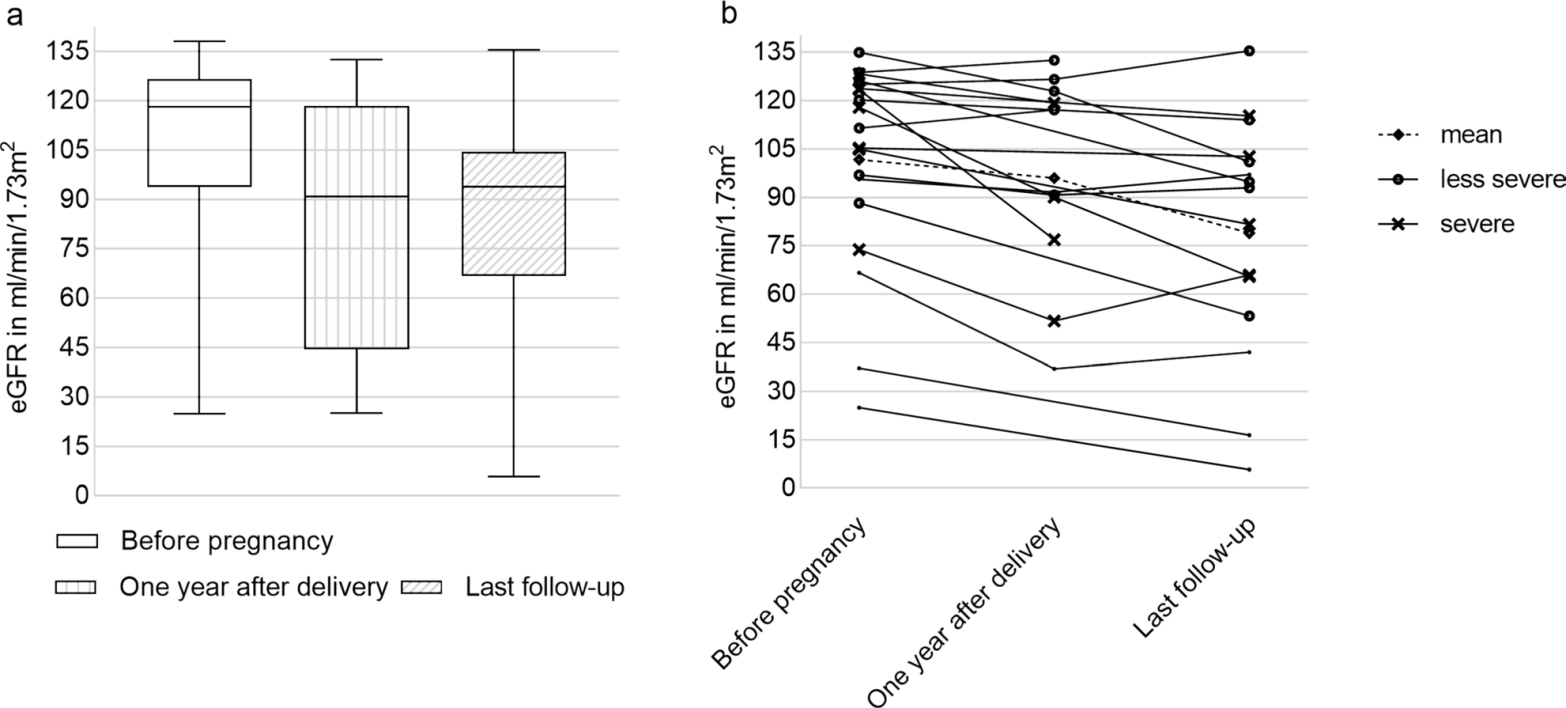
Course of eGFR before and after pregnancy. **a** eGFR before pregnancy (*n* = 22; 105.4 ± 30.7 ml/min/1.73 m^2^), one year after delivery (*n* = 17; 86.9 ± 36.7 ml/min/1.73 m^2^) and at last follow-up (*n* = 32; 85.0 ± 29.5 ml/min/1.73 m^2^; median 5 years, range 1–27 years after delivery). **b** Intra-individual course of eGFR, paired samples, Wilcoxon test (p < 0.05)

The eGFR data of 22 women (17 with heterozygous X-linked Alport syndrome, two with autosomal recessive Alport syndome, and 2 unknown) before pregnancy and 32 women (27 with heterozygous X-linked, two with autosomal recessive Alport syndome, and 3 unknown) after pregnancy were available (Fig. 2a). For paired samples, compared to those prior to pregnancy, the mean eGFR at one year after delivery decreased significantly from 109.3 ± 23.1 to 96.0 ± 31.4 ml/min/1.73 m^2^ (*n* = 11; *p* = 0.021). At last follow-up, the mean eGFR was lower than that before pregnancy, which again decreased significantly from 96.1 ± 32.9 to 78.9 ± 37.0 ml/min/1.73 m^2^ (*n* = 15; *p* = 0.003), resulting in a mean eGFR loss of 17.2 ± 16.7 ml/min/1.73 m^2^ at a mean follow-up time of 3.7 ± 2.0 years after delivery (Fig. 2b).

Of the 15 women with X-linked Alport syndrome, 8 (50%) had less severe variants, while 7 (50%) had severe variants. Mean eGFR loss was higher in those with severe variants (*n* = 7) compared to individuals with less severe variants (*n* = 8) (– 21.5 ± 20.2 vs. – 11.3 ± 19.0 ml/min/1.73 m^2^; Fig. 2b). In addition, eGFR loss was higher in women with already impaired renal function (defined as eGFR < 90 ml/min/1.73 m^2^) (*n* = 5) compared to women with normal renal function (*n* = 14) (– 21.5 ± 9.8 vs. – 14.0 ± 20.0 ml/min/1.73 m^2^; Fig. 2b).

In addition, two cases of women with impaired renal function before pregnancy are described further:

A 24-year-old woman with autosomal recessive Alport syndome had a creatinine level of 2.6 mg/dl (eGFR 25 ml/min/1.73 m^2^), when she became pregnant. At week 28 of gestation, creatinine started to increase, and fetal growth retardation was detected. Preeclampsia was diagnosed, placental insufficiency was suspected and lung maturation was induced. At 33 weeks of gestation, a cesarean section was performed, and a preterm infant weighing 1400 g was born. The infant had respiratory adaptation disorder. The patient's creatinine increased to 5.6 mg/dl after delivery. Approximately one year after delivery, hemodialysis was started (creatinine 8.6 mg/dl; eGFR 5.8 ml/min/1.73 m^2^; blood urea: 122 mg/dl). Five years later, an allogeneic kidney transplant was performed. Graft failure and the need for dialysis occurred twelve years after kidney transplantation.

The second case regards a 35-year-old woman with autosomal recessive Alport syndome with impaired renal function (creatinine 1.7 mg/dl; eGFR of 36 ml/min/m^2^; proteinuria 3.3 g/day) prior to pregnancy and acute appendicitis during pregnancy (third trimester). A single intra-partum dialysis session due to hyperkalemia was required. Due to nephrotic syndrome with hypertensive blood pressure (165/100 mmHg) a cesarean section was performed at 33 weeks of gestation. The newborn weighed 1800 g. Postpartum, the mother had peripartum cardiomyopathy, which was treated with bromocriptine. Hemodialysis due to hypervolemia with lung edema had to be performed. Following the recovery period, the patient's creatinine level was 3.1 mg/dL (eGFR 18 ml/min/m^2^), and renal function remained stable for three years. Subsequently, she underwent a living-donor kidney transplant.

### Disease stages of Alport syndrome prior to, during, and after pregnancy

**Fig. 3:**
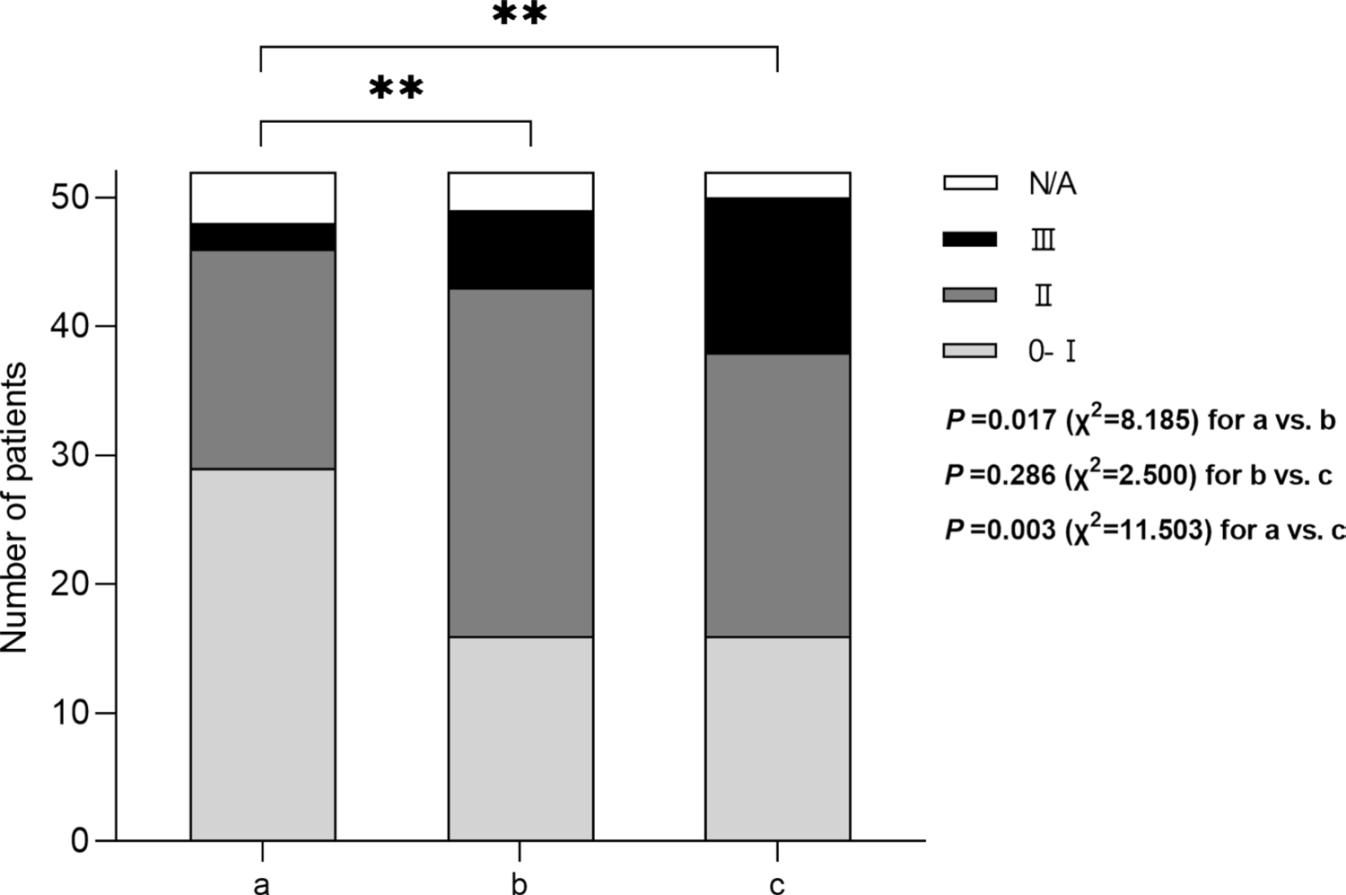
Disease stages in pregnant women with Alport syndrome. **a** Alport stages prior to pregnancy (*n* = 52); **b** Alport stages during pregnancy (*n* = 52); **c** Alport stages after pregnancy (*n* = 52)

**Fig. 4:**
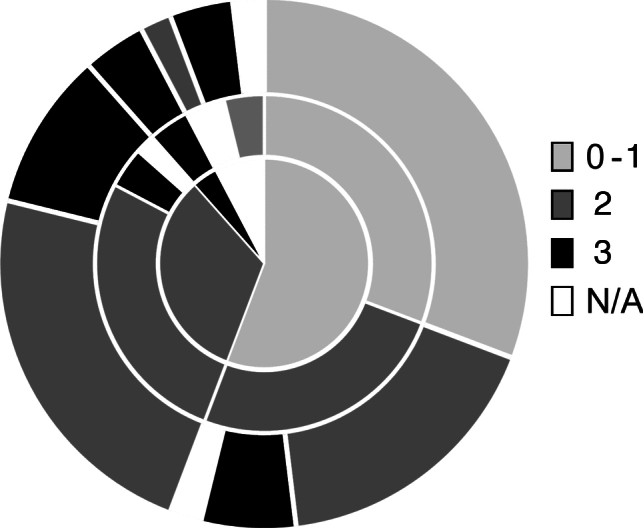
Visualization of disease progression of Alport patients in the various Alport stages prior to pregnancy. The central area shows the composition of Alport stages before pregnancy (stage 0-1 *n* = 29, stage 2 *n* = 17, stage 3 *n* = 2, N/A *n* = 4); the middle area shows the disease stage during pregnancy (stage 0-1 *n* = 16, stage 2 *n* = 27, stage 3 *n* = 6, N/A *n* = 3); and the outer ring shows disease stage after delivery (stage 0-1 *n* = 16, stage 2 *n* = 22, stage 3 *n* = 12, N/A *n* = 2)

Disease stages prior to, during, and after pregnancy were evaluated in 52 women with Alport syndrome (50 with X-linked and 2 with autosomal recessive Alport syndome). The distribution of disease stages before and after pregnancy are shown in Fig. 3. Disease progression is shown in Fig. 4. Altogether, 42% (22/52) of women had a progression of Alport stage, 31% (16/52) remained in stage 0-1 and 23% (12/52) remained in stage 2 after pregnancy.

### Treatment prior to pregnancy and after delivery

Before pregnancy, seven patients received angiotensin-converting enzyme inhibitor (ACEi) or angiotensin receptor blocker (ARB) therapy. Most patients (6/7) discontinued the drugs 2–4 months prior to pregnancy, the one remaining patient stopped in the fourth week of pregnancy. No fetal malformations were observed.

After delivery, twelve patients received nephroprotective therapy, specifically 8 with ACEi/ARBs, one with sodium-dependent glucose transporter 2 inhibitor (SGLT2i), and one with combined treatment of ACEi, ARB and SGLT2i. Medication was unknown for two other patients. Treatment was started 11 months (range 1–38 months) after delivery and proteinuria decreased from 1.8 ± 1.3 to 0.8 ± 0.8 g/day. In contrast, 76.9% (40/52) of patients remained off therapy after delivery, despite the fact that many had overt proteinuria.

## Discussion

In this study, the progression of chronic kidney disease was retrospectively evaluated in pregnant women with Alport syndrome from China and Europe: 42% of women experienced progression of their kidney disease after delivery. Increased proteinuria and decreased eGFR after delivery were observed, especially in women with severe variants or eGFR < 90 ml/min/1.73 m^2^ prior to pregnancy.

The mean eGFR declined from 109.3 ± 23.1 to 96.0 ± 31.4 ml/min/1.73 m^2^ at one year after delivery. At a mean follow-up time of 3.7 ± 2.0 years after delivery, eGFR further declined to 78.9 ± 37.0 ml/min/1.73 m^2^. This is partially different from a recent report of pregnancies in women with COL4A3-5-related disease [26], which showed that mean eGFR decreased after pregnancy but remained within normal range. This might be due to differences in population and postpartum follow-up periods. In the future, prospective studies or analyses of eGFR slope are needed to evaluate the progression of renal function before and after pregnancy.

In our study, most women with Alport syndrome still had normal renal function after pregnancy. Altogether, 23.6% (13/55) of included patients experienced worsening of kidney function after pregnancy (eGFR declined to below 90 ml/min/1.73 m^2^ or further decline). We reviewed 24 previously published case reports and found that 38% (9/24) experienced worsening of kidney function during pregnancy [18, 20, 27–29] [S3-S10] (Supplementary Table 1).  

One new finding of our study is that the severity of pathogenic variants and eGFR < 90 ml/min/1.73 m^2^ prior to pregnancy have a higher risk of postpartum eGFR loss in women with Alport syndrome. A previous case report [20] supported that low eGFR and higher proteinuria prior to pregnancy predict worsening of kidney function during and after pregnancy. Therefore, the type of variant and low eGFR before pregnancy could be considered relevant risk factors, but larger studies need to confirm these findings. Additionally, previous studies identified high blood pressure as a risk factor for postpartum kidney failure [S11].

The amount of albuminuria or proteinuria is an indicator of severity of disease progression in non-pregnant women with X-linked Alport syndrome [14, 30]. In our study, the level of proteinuria after pregnancy increased significantly. The number of women with Alport stage II (overt proteinuria) increased to 50% (26/52) during pregnancy, and to 42.3% (22/52) after pregnancy, compared to 32.7% (17/52) prior to pregnancy. Previous reports in a small number of cases suggested that increased proteinuria can persist or even progress after delivery in women with Alport syndrome [26–29]. Unfortunately, our study could not determine whether the increase in proteinuria after pregnancy was related to the lack of nephroprotective therapy, and further research is needed.

In our study, the majority of women with Alport syndrome had an uncomplicated pregnancy, and the complicated pregnancies (22.6%) were lower than in the previous studies [29].

The limitations of our study are the small sample size, the presence of missing values, and the bias of the retrospective study design. The absence of measured proteinuria and eGFR data in a subset of patients suggests that these patients had less severe CKD compared to those with available data. This potential selection bias should be considered when interpreting our findings. In the future, age-matched non-pregnant female Alport syndrome patients could serve as a control group to analyze the effect of pregnancy on the kidney outcomes in women with Alport syndrome.

In conclusion, our study provides further data on the natural history of Alport syndrome in women who have had a pregnancy. Our findings show that most women with Alport syndrome with normal eGFR prior to pregnancy experience an uncomplicated pregnancy and have good kidney function after pregnancy. In contrast, women with severe variants and with eGFR < 90 ml/min before pregnancy are at higher risk of decline in eGFR after delivery. Further studies are needed to improve outcome of pregnancies in women with Alport syndrome. 

## Supplementary Information

Below is the link to the electronic supplementary material.

Supplementary file1 (DOCX 18 KB)

## Data Availability

Original data will be made available upon request.
