# Supplementary material for: A retrospective study of kidney disease in Alport syndrome during and after pregnancy
Source: J Nephrol. 2025 May 20;38(3):1085–92. doi: 10.1007/s40620-025-02252-2 (PMC12165875; doi:10.1007/s40620-025-02252-2)
Supplement: Supplementary file 1 — Supplementary file1 (DOCX 18 KB) [file 40620_2025_2252_MOESM1_ESM.docx]

**Supplemental Table 1**. Renal damage of published cases in pregnant women with Alport syndrome

| Author, Year, Reference | Patient Number | Before or early pregnancy | |  | During pregnancy | | | |  | After pregnancy | | | |
| --- | --- | --- | --- | --- | --- | --- | --- | --- | --- | --- | --- | --- | --- |
|  |  | Serum creatinine (mg/dL) | Creatinine clearance rate (ml/min) |  | weeks of gestation | Serum creatinine (mg/dL) | Creatinine clearance rate (ml/min) | eGFR (ml/min/1.73 m^2^) |  | Months after delivery | Serum creatinine (mg/dL) | Creatinine clearance rate (ml/min) | eGFR (ml/min/1.73 m^2^) |
| Matsuo K TE, 2007 | 1 | 1.0 –1.2 | 57.8 |  | 25 | 7.3 |  |  |  |  | 8.3 |  |  |
| Matsubara S, 2009 | 2 | 0.59 |  |  | 31 | 0.54–0.70 | 97.2–121.9 |  |  | 6 | 0.5–0.7 |  |  |
| Mehta S, 2013 | 3 | 0.7 |  |  | 29 | 1.53 |  |  |  | Second day after delivery | 1.1 |  |  |
| Alessi M, 2013 | 4 | normal |  |  | 30 |  | 86.3 |  |  | 22 | 0.9 | 81.7 |  |
|  | 5 | normal |  |  |  |  |  |  |  | 22 | 1.48 | 42 |  |
| Crovetto F, 2013 | 6 | normal |  |  | 21 | 0.51 |  |  |  | 5 | 0.58 |  |  |
|  | 7 | 0.54 |  |  | 28 | 0.53 |  |  |  | 6 | 0.61 |  |  |
| Nishizawa Y, 2015 | 8 | 0.62 |  |  | 36 | 0.48 |  |  |  | 2 | 0.47 |  |  |
| Yefet E, 2016 | 9 | N/A |  |  |  |  |  |  |  |  |  |  |  |
|  | 10 | N/A |  |  |  | Worsen |  |  |  |  |  |  |  |
|  | 11 | 0.58 |  |  | 40 | 0.9 |  |  |  |  |  |  |  |
| Kitanovska BG, 2016 | 12 | N/A |  |  | 29 | 120-150 micromol/l |  |  |  | 24 | 70-83 micromol/l |  |  |
| Brunini F, 2018 | 13 | 0.6 |  |  |  | 0.8 |  |  |  |  | 0.7 |  |  |
|  | 14 | 0.65 |  |  |  | 0.8 |  |  |  |  | 0.83 |  |  |
|  | 15 | 0.7 |  |  |  | 0.5 |  |  |  |  | 0.7 |  |  |
|  | 16 | 0.54 |  |  |  | 0.57 |  |  |  |  | 0.61 |  |  |
|  | 17 | 0.8 |  |  |  | 1.12 |  |  |  |  | 1.2 |  |  |
|  | 18 | 2.4 |  |  |  | 4.4 |  |  |  |  | HD |  |  |
| Drury ER, 2019 | 19 | 0.77 |  |  |  | 1.09 |  |  |  | 10 | 0.9 |  |  |
| Pepe F, 2020 | 20 | N/A |  |  |  | Worsen |  |  |  |  | Recovered |  |  |
| Shi WH, 2021 | 21 | normal (<0.7) |  |  |  | normal (<0.7) |  |  |  |  | normal (<0.7) |  |  |
|  | 22 | normal (<0.7) |  |  |  | normal (<0.7) |  |  |  |  | normal (<0.7) |  |  |
|  | 23 | normal (<0.7) |  |  |  | normal (<0.7) |  |  |  |  | normal (<0.7) |  |  |
| Jehn U, 2022 | 24 | N/A |  |  | 29 | 1.4 |  | 51 |  | 3 |  |  | 37 |

**Supplemental References**

S1. Wiles, K., K. Bramham, P.T. Seed, et al., *Serum Creatinine in Pregnancy: A Systematic Review.* Kidney International Reports, 2019. **4**(3): p. 408-419.

S2. Dvořák, J., M. Koucký, E. Jančová, et al., *Chronic kidney disease and pregnancy outcomes.* Scientific Reports, 2021. **11**(1).

S3. Matsubara, S., Y. Ueda, H. Takahashi, et al., *Pregnancy complicated with Alport syndrome: a good obstetric outcome and failure to diagnose an infant born to a mother with Alport syndrome by umbilical cord immunofluorescence staining.* J Obstet Gynaecol Res, 2009. **35**(6): p. 1109-14.

S4. Mehta, S., C. Saifan, M. Abdellah, et al., *Alport's Syndrome in Pregnancy.* Case Rep Med, 2013. **2013**: p. 374020.

S5. Alessi, M., A. Fabris, A. Zambon, et al., *Pregnancy in Alport syndrome: a report of two differently-evolving cases.* J Obstet Gynaecol, 2014. **34**(1): p. 98-100.

S6. Crovetto, F., G. Moroni, B. Zaina, et al., *Pregnancy in women with Alport syndrome.* Int Urol Nephrol, 2013. **45**(4): p. 1223-7.

S7. Nishizawa, Y., T. Takei, T. Miyaoka, et al., *Alport syndrome and pregnancy: Good obstetric and nephrological outcomes in a pregnant woman with homozygous autosomal recessive Alport syndrome.* J Obstet Gynaecol Res, 2016. **42**(3): p. 331-5.

S8. Brunini, F., B. Zaina, D. Gianfreda, et al., *Alport syndrome and pregnancy: a case series and literature review.* Arch Gynecol Obstet, 2018. **297**(6): p. 1421-1431.

S9. Pepe, F., F. Di Guardo, E. Zambrotta, et al., *Renal impairment in Alport syndrome pregnant woman: Case report and review of the literature.* Clin Case Rep, 2020. **8**(12): p. 3003-3007.

S10. Jehn, U., C. Muller-Hofstede, B. Heitplatz, et al., *A Novel Homozygous Mutation in the COL4A4 Gene (Gly1436del) Causing Alport Syndrome Exposed by Pregnancy: A Case Report and Review of the Literature.* Case Rep Nephrol, 2022. **2022**: p. 5243137.

S11. Wu, C.-C., S.-H. Chen, C.-H. Ho, et al., *End-stage renal disease after hypertensive disorders in pregnancy.* American Journal of Obstetrics and Gynecology, 2014. **210**(2): p. 147.e1-147.e8.
